# Empowering communities and strengthening systems to improve transgender health: outcomes from the Pehchan programme in India

**DOI:** 10.7448/IAS.19.3.20809

**Published:** 2016-07-17

**Authors:** Simran Shaikh, Gitau Mburu, Viswanathan Arumugam, Naveen Mattipalli, Abhina Aher, Sonal Mehta, James Robertson

**Affiliations:** 1 India HIV/AIDS Alliance, New Delhi, India; 2 International HIV/AIDS Alliance, Brighton, UK; 3 Department of Health Research, Lancaster University, Lancaster, UK

**Keywords:** Transgender, Hijra, Community, HIV, India

## Abstract

**Introduction:**

Transgender populations face inequalities in access to HIV, health and social services. In addition, there is limited documentation of models for providing appropriately tailored services and social support for transgender populations in low- and middle-income countries. This paper presents outcomes of the Global Fund-supported Pehchan programme, which aimed to strengthen community systems and provide HIV, health, legal and social services to transgender communities across 18 Indian states through a rights-based empowerment approach.

**Methods:**

We used a pre- and post-intervention cross-sectional survey design with retrospective analysis of programmatic data. Using stratified sampling, we identified 268 transgender participants in six Indian states from a total of 48,280 transgender people served by Pehchan through 186 community-based organizations. We quantified the impact of interventions by comparing baseline and end line indicators of accessed health social and legal services. We also assessed end line self-efficacy and collective action with regard to social support networks.

**Results:**

There were significant increases in community-based demand and use of tailored health, legal, social and psychological services over the time of the Pehchan programme. We report significant increases in access to condoms (12.5%, *p*<0.001) and condom use at last anal sex with both regular (18.1%, *p*<0.001) and casual (8.1%, *p*<0.001) male partners. Access to HIV outreach education and testing and counselling services significantly increased (20.10%, *p*<0.001; 33.7%, *p*<0.001). In addition, significant increases in access to emergency crisis response (19.7%, *p*<0.001), legal support (26.8%, *p*<0.001) and mental health services (33.0%, *p*<0.001) were identified. Finally, we note that the Pehchan programme successfully provided a platform for the formation, collectivization and visibility of peer support groups.

**Conclusions:**

The Pehchan programme's community involvement, rights-based collectivization and gender-affirming approaches significantly improved both demand and access to tailored HIV, health and social services for transgender individuals across India. Furthermore, the Pehchan programme successfully fostered both self-efficacy and collective identity and served as a model for addressing the unique health needs of transgender communities. Continued strengthening of health, social and community systems to better respond to the unique needs of transgender communities is needed in order to sustain these gains.

## Introduction

Transgender populations in low-, middle- and high-income countries face inequality of access to HIV and health services [1]. In India, the estimated prevalence of HIV at the national level is 0.31% [2], whereas HIV prevalence in the transgender population is estimated to be 8.2% [2]. While the HIV prevalence and incidence data among transgender populations differ, a constellation of factors at the individual, community and structural levels determine these patterns.

At the individual level, and similar to other populations, unprotected sexual exposure and sex work, as well as multiple casual sexual partners, can contribute to HIV risk [3,4]. Hormone injecting has also been associated with HIV exposure among transgender populations [1]. Similar to other populations, transgender people often lack adequate knowledge regarding HIV transmission [5,6] and in India, they may also have high levels of sexually transmitted infections (STIs) that could contribute to HIV transmission [7].

At the interpersonal and community levels, transgender populations often experience high levels of both perceived and internalized social stigma [8,9], social isolation, discrimination and victimization [10]. Extreme social exclusion and lack of acceptance of transgender populations in different settings diminishes their self-esteem and ability to participate in social events [11]. These situations often lead to symptomatic psychological distress, depression, anxiety and other mental health difficulties among this population [9,12–14]. Social victimization may occasionally contribute to poor sexual health and unhealthy use of alcohol among this group, for example in India [10].

Structural factors also contribute to poor health and HIV risk among transgender populations. Healthcare for transgender populations is limited due to stigma among health professionals [15] and within health facilities [16]. In many contexts, medical training excludes transgender health [17] and, as a result, health professionals lack the appropriate skills and competencies to provide tailored services to transgender populations [17,18].

In India, poverty and economic exclusion contribute to livelihood deprivation among transgender persons [19], and prevent their access to sex-reassignment surgery for gender transition and other healthcare services [20,21]. As a result, an estimated 20% of the transgender population in India has unmet transgender-specific healthcare needs [22]. Additionally, economic marginalization has also meant that 20 to 30% of transgender populations in India engage in begging or sex work as their primary occupation [21]. Yet criminalization of sex work combined with aggressive policing has often resulted in violence directed towards this population in India and globally [21,23].

While the provisions for a “third gender” status made in a 2014 Supreme Court ruling may have been perceived as securing transgender rights, a third gender status is not universally accepted across India. Furthermore, legislative discrimination persists, as transgender rights remain suppressed by Section 377 of the Indian Penal Code, which criminalizes same-sex relations [24]. Yet there is an urgent need for a rights-based approach to address HIV and health needs among the transgender population [1], especially in light of the current global [25] and Indian [26] commitment to achieve sustainable control of the HIV epidemic.

To this end, it is critical to identify ways in which HIV services in India can be readjusted to adequately address the needs of the transgender population. Yet there is very limited documentation on effective models for providing tailored health, social and legal protection services to transgender populations, especially in low- and middle-income settings [27]. Nevertheless, aspects of models from high-income settings could be replicated at the community level in low- and middle-income settings. For instance, in the United States, two studies demonstrated that community-based outreach, social inclusion and peer support successfully mitigated social stigma and psychological distress and generally improved health outcomes among transgender individuals [9,13]. This paper documents outcomes from the Pehchan programme, which has been providing HIV, health, legal and social protection services to transgender communities across 18 Indian states through a rights-based and empowerment approach.

## Methods

### Intervention origin and description

In India, the Pehchan programme aims to increase access to health, social and legal services through community-based and peer-led social support systems that encourage gender-affirming empowerment. Pehchan serves the men who have sex with men (MSM), transgender and *hijra* communities. In this study, *transgender* is used an umbrella term that also includes hijra persons, a South Asian subgroup of the transgender community. In Hindi, *pehchan* means “identity,” “recognition” or “acknowledgement.” The origin of the programme dates back to March 2008 when a group of eight Indian community activists – including one transgender and one hijra – met to discuss the opportunity for a large-scale programme by and for their own communities to complement the Indian government's services. Pehchan is financed by the Global Fund to Fight AIDS, Tuberculosis and Malaria and is the largest single-country global fund grant focusing on vulnerable and underserved sexual minorities to date. The programme began in October 2010 and has strengthened the capacity of 200 community-based organizations (CBOs) to provide tailored HIV services. By August 2015, the programme had reached more than 433,000 community members, 60% of whom had never been reached by HIV prevention services before. The programme itself was developed through a process of consultation involving a consortium of partners, community leaders, the government of India's National AIDS Control Organization (NACO) and State AIDS Control Societies, and the Global Fund, among other stakeholders. The India HIV/AIDS Alliance was chosen as the principal recipient (PR) by the leaders of transgender communities and has worked closely with the programme's regional sub-recipients (SRs) – Humsafar Trust, Pehchan North Region Office, SAATHI, SIAAP, Sangama and AIAP – and 200 sub-sub-recipients (SSRs) spread over the 18 states to implement the programme.

### Specific components and activities

Pehchan provides three broad categories of activities. The first category of activities focuses on improving the organizational and technical capacity of CBOs working with transgender communities. Using a capacity-building approach [28], the programme strengthens the community mobilization, leadership, programming, planning, monitoring and budgeting capacity of CBOs, enabling them to participate more effectively in the delivery of targeted interventions as per the national guidelines, to prevent HIV among high-risk groups and support those who are HIV positive to get linked to care. By helping build strong CBOs, Pehchan addresses capacity gaps that often prevent such organizations from effectively contributing to community-based HIV prevention in many contexts [29,30]. The second category of activity is support to CBOs to provide a range of basic community-based prevention and linkage to care interventions, as defined by and as defined by India's National AIDS Control Program. To ensure that the programme was sensitive to the needs of transgender persons, the programme facilitated the participation of transgender representatives at every level. For instance, a transgender professional was leading the programme as national manager, and transgender persons were recruited as staff across organizations serving as PR, SR and almost all SSRs. In addition, transgender persons were engaged in preparing and delivering all the capacity building, research and communication activities, alongside other subject matter experts. This way, transgender communities defined what services were most needed and in what way they should be provided to transgender communities.

These interventions include behavioural change communication such as outreach-based interpersonal communication through peers, educational materials, closed Facebook page, media posters and facility-based one-on-one counselling. These communications provide counselling and information on safe sex, STIs, condom use, HIV testing and, in addition, ART adherence to transgender individuals living with HIV. The third category relates to the creation of a supportive environment for transgender communities. This extended package of services includes legal support, support for accessing social entitlements, identity, mental health and psychosocial counselling, relationship counselling, life skills, community-based group mobilization and empowerment, advanced crisis and trauma management, family support and counselling related to sexual and reproductive health.

### Theoretical basis of intervention

This intervention was based on a self-efficacy framework. Self-efficacy is a concept that captures people's beliefs and actual ability to thrive in different, potentially difficult social situations. It can be improved based on a person's experiences and gained competencies [31]. It has therefore been employed by scholars such as Cicognani [32] and Tsang [33] to understand the processes through which empowerment and collective bargaining power can be enhanced so as to enable people to overcome social stressors and have better control and influence over their own circumstances. Community mobilization, collectivization and empowerment strategies have been shown to empower marginalized sexual minorities [34–36].

While community mobilization is often a loosely applied term, authors emphasize that in this programme it encompassed wider principles of involvement in or influence on the design, implementation and quality monitoring of the programme, and not merely a local grouping of marginalized transgender communities. More specifically, and in this project, the concept of community involvement was operationalized through staff recruitment policies, engagement of communities in technical areas and in a feedback mechanism though a community advisory board. The community advisory board included transgender representatives who were not directly employed by the programme, to provide quarterly feedback regarding Pehchan services. This concept recognizes that health experiences and outcomes are often influenced by the ability of individuals to demand services, which is in turn influenced by self-efficacy and collective action to overcome social and structural barriers to services.

### Evaluation methodology

#### Design

To evaluate the impact of the Pehchan interventions, we used a pre- and post-intervention cross-sectional design with retrospective analysis of programmatic data. We conducted an end line survey in 2015 and compared the findings to those of the baseline survey conducted in 2011, the findings of which have been reported elsewhere [37,38]. Both the baseline and end line surveys were similar in content and followed similar protocols in participant recruitment but did not include the exact same participants. We adopted a quantitative design to track changes in key indicators over time.

#### Sampling procedure

We employed a stratified systematic random sampling to identify 268 transgender participants from a total population of 48,280 transgender clients in the six states (covered by 112 CBOs): Andhra Pradesh and Telangana, Karnataka, Maharashtra, Tamil Nadu, Uttar Pradesh and West Bengal. Sample size was calculated to detect a minimum required difference in key indicators between baseline and end line surveys at 80% power and a 95% two-sided confidence level of 0.05. We accounted for a design effect of 1.5 for multistage sampling and estimated 5% for no response or invalid responses. A primary programme indicator on which the sample size was calculated was a 4% increase in the percentage of respondents using condoms during last sex with men. We stratified based on gender identity and coverage in programme sites; to capture differences among minority transgender populations, we determined that at least 30% of the respondents had to be transgender individuals.

We determined the extent of Pehchan coverage by assessing the numbers of transgender people accessing services from a comprehensive list of CBOs in each state. We then used a probability proportional to size sampling method to determine the required number of districts to include in our study. This sampling method reduces bias by ensuring that the probability of selecting a district is proportional to its contribution [39] and is superior to simple random sampling with equal probability as it results in smaller variances [39,40]. In total, we included 33 districts and selected potential participants using systematic random sampling from a list of clients being served by the CBOs. Study participants were included if they had been registered in the programme for at least six months, were willing to provide written consent to participate voluntarily, had had anal sex with a male partner in the last three months and were over 18 years of age.

#### Study tools and measures

While our end line structured questionnaire retained key elements of the baseline questionnaire [37,38], we expanded the questions on community mobilization and sexual behaviours and introduced questions on programme exposure and collective efficacy. The end line questionnaire focused on the sociodemographic characteristics of respondents, their sexual behaviour including condom use patterns, access and results of HIV testing services, experiences of stigma and discrimination, awareness of Indian Penal Code Section 377, instances of abuse, community and police violence or harassment, collective action and efficacy, exposure to the Pehchan programme, awareness and access of intervention services and biological feminization. Representatives from the CBOs were involved in finalizing the study protocols and preparing research tools and fieldwork plans, as well as field data collection. The structured questionnaire had both open-ended and closed questions to capture qualitative information, as well as built-in checks to ensure the reliability and validity of the data.

Questionnaires were translated into six local languages (Bengali, Hindi, Kannada, Marathi, Tamil and Telugu) by local research assistants, using a standard forward-backward procedure to preserve intended meanings and cultural appropriateness. The translated versions were used for a three-day training of field teams in the study states, which covered the study objectives, methodology, meanings of the terms used in the study, confidentiality, questionnaire explanation, and recording and validating of questionnaire responses. Translations and question routing were assessed during piloting and amendments were made.

#### Data collection and management

We used accompaniment and spot checks in the field to assure the quality of data collection. In accompaniment, the supervisor attended the interview to observe that the interviewer was asking questions exactly as written and in the same sequence. In the spot checks, the supervisors and the field-based project manager visited the interview site to ensure that interviewers were conducting interviews in identified secure locations and with the selected respondents following the fieldwork plan. Data were checked for completeness and logic to minimize data entry errors.

#### Questionnaire interview procedures

Participants were approached at the CBOs, informed of the purpose of the survey and invited to participate. After consenting, the questionnaire was administered to respondents face-to-face by local trained researchers. In all cases, interviews were conducted in a private room at the CBO or at drop-in centres operated by the CBOs. Each interview lasted 45 to 60 minutes. To complement survey data, we used tally records of services provided by CBOs to extract relevant programme data.

### Data analysis

Survey and CBO programme data were manually checked for completeness and entered into CSPro software, which has built-in scrutiny checks to minimize data entry errors. These data were then electronically imported into SPSS v17.0 for analysis. We used descriptive analyses to examine participant characteristic and service access and association analyses to assess the differences between baseline and end line findings.

### Ethical considerations

During this study, we used appropriate approaches for obtaining informed consent and safeguarding privacy and confidentiality [41]. In particular, the researchers explained the purpose of the study; participants were informed of their right to end the interviews at any stage and were provided with the contact information of the principal investigators for questions or concerns. The researchers were trained to conduct interviews with respect for the dignity of the respondents. Apart from travel expenses and light refreshments during the interviews, no incentives were provided. Participants were specifically recruited through CBOs already working with sexual minorities, which mitigated potential concerns related to personal identification and stigma. All personally identifiable data were deleted. Ethical approval was obtained from the Sigma Institutional Review Board (ref: 10004/IRB/D/15–16).

## Results

### Survey participant characteristics

A total of 268 transgender participants were included in the end line survey. The mean age was 28 years of age, 20% were involved in sex work and 27%, mainly hijras, were living communally (Table 1). These characteristics are compared with the findings from transgender participants in the baseline survey, which have been reported elsewhere [37,38].

**Table 1:** Characteristics of transgender participants in the baseline and end line surveys

	Baseline (*n*=277)		End line (*n*=268)			
				
Characteristic	*n*	%	*n*	%	χ^2^ test statistic	*p*
Mean age (years)	30.05±9.4		28.45±7.3		−2.282(t)	0.023
Education						
Illiterate	57	21	47	18	0.82	0.37
Primary school	39	14	91	34	29.63	<0.001
Secondary school	146	53	108	40	8.43	<0.001
Graduate and above	35	13	22	7	2.85	0.09
Vocational training	0	0	58	22	–	–
Main occupation						
Salaried job	32	12	28	10	0.17	0.68
Unemployed	9	3	58	21	46.97	<0.001
Student	14	5	12	4	0.1	0.75
Labourer	5	2	31	11	21.04	<0.001
Self-employed	15	5	7	3	2.76	0.10
Other trade	3	1	9	4	3.27	0.07
Dancer	83	30	30	11	29.2	<0.001
Sex work	153	55	53	20	72.84	<0.001
Other	11	4	8	4	0.39	0.53
Monthly income (in INR)						
Less than 3000	82	31	57	22	23.5	<0.001
3001 to 6000	104	39	77	30		
6001 to 10,000	39	15	79	30		
More than 10,000	37	14	48	18		
Mobile phone ownership	–	–	244	91%		
Immigrant	213	77	62	24%	157.49	<0.001
HIV testing status	220	79	257	96	33.85	<0.001
Living situation						
Living alone	70	25	54	20	2.03	0.15
Cohabiting	37	13	15	6	9.5	<0.01
Living with spouse	0	0	4	1	–	–
Living with parents	48	17	113	42	40.37	<0.001
Communal	106	38	77	27	5.55	0.02
Living with relative	4	1	10	4	2.85	0.09
Substance use						
Alcohol	178	64	147	55%	5.01	0.03
Tobacco	22	8	117	44%	91.45	<0.001
Stimulants	0	0	13	5%	–	–
Opioids	4	1	3	1%	0.11	0.74

INR, Indian rupee.

### Increased access and use of tailored sexual and reproductive health services

All measures of access to and use of sexual and reproductive health services indicate significant increased access and use from baseline to end line (Table 2). Through July 2015, 9587 transgender clients, including 4028 hijra individuals, had been provided with STI treatment, an increase of 47.8% since baseline. Overall, 1,977,932 condoms were provided to transgender and 1,593,721 to hijra clients through outreach-based distribution and access to condoms and condom use increased during this time. Finally, the data demonstrate a significant increase in access to information and services related to sex reassignment, such as hormone use and surgery.

**Table 2:** Access to sexual and reproductive health services

Indicator	Baseline, % (*n*=277)	End line, % (*n*=268)	Net change, %	χ^2^ test statistic	*p*
Access to condoms	82.3	94.8	12.5	20.7	<0.01
Condom use during last anal sex (regular male partner)	70.1	89.0	18.1	30.57	<0.01
Condom use at last anal sex (non-regular male partner)	84.4	92.5	8.1	8.66	<0.01
Access to lubricants	61.4	80.4	19.0	23.34	<0.001
Access to STI treatment	31.0	78.8	47.8	124.9	<0.001
Sexual reproductive health services for female partners	15.5	48.6	33.0	68.4	<0.001
Sex reassignment surgery information	30.3	61.7	31.3	53.58	<0.001

STI, sexually transmitted infection.

### HIV testing and counselling

Our data indicate that the Pehchan programme increased the proportion of transgender individuals reached with HIV-related education, referred to testing and provided with ART adherence counselling (Table 3). HIV testing in transgender communities increased 33.7% (*p*<0.001) over the study period and, by July 2015, a total of 34,087 for transgender clients, including 11,547 hijra were referred to counselling and testing services and 48,280 transgender clients, including 16,015 hijra, received outreach-based education for behavioural changes that reduce HIV risk. In the end line survey, almost all participants were offered HIV education (94.8%) and had been referred for HIV testing and counselling (93.9%). A total of 28,493 HIV tests were provided to transgender and hijra clients and 583 clients were identified as seropositive.

**Table 3:** Comparison in HIV testing between baseline and end line surveys

Indicator	Baseline, % (*n*=277)	End-line, % (*n*=268)	Net change, %	χ^2^ test statistic	*p*
Reached with HIV education through outreach	74.7	94.8	20.1	41.99	<0.001
Referred for HIV testing and counselling	59.2	93.9	33.7	91.43	<0.001
Receiving ART adherence counselling if on ART	32.9	41.8	7.9	4.66	0.297

### Increased access to legal and psychological services and advocacy training

Due to high levels of arrest, extortion, violence and harassment experienced by the transgender population [38], interventions to mitigate these experiences were a central part of Pehchan. Demand for legal and psychological services was high and increased over the survey time period (Table 4). By July 2015, 48,280 transgender clients had used services related to the emergency crisis response, legal support, and empowerment and advocacy training to empower and increase self-advocacy skills. Services used also included counselling for those with symptoms of depression, anxiety or post-traumatic stress following violence, police arrests or other harassment. We also noted a significant increase in access to drop-in centres, which play a particularly important role by bringing the programme beneficiaries together, creating opportunities to share problems, collaborate on potential solutions, and form alliances for collective action.

**Table 4:** Access to legal, emergency and psychological services

Indicator	Baseline, % (*n*=277)	End line, % (*n*=268)	Net change, %	χ^2^ test statistic	*p*
Access to emergency crisis response	48.7	68.4	19.7	19.08	<0.001
Access to legal support	38.9	65.7	26.8	33.17	<0.001
Empowered with advocacy skills for services	42.9	77.9	35.0	59.54	<0.001
Access to mental health support	43.7	77.2	33.0	53.95	<0.001
Access to drop-in and safe centres	57.0	76.9	19.9	24.04	<0.001

### Access to social protection

In relation to social protection, the programme facilitated the provision of various social entitlements, documents and amenities that are provided by the government, such as a ration card (a document needed for accessing food rations from the public distribution system), Aadhaar card (a newly introduced identity card also used for many bank and government benefits), benefits card for those below the poverty line, bank account, voter identity card, Permanent Account Number Card, passport and social insurance. Survey data showed that the programme improved access to these entitlements, although data related to whether these entitlements were provided with their names given at birth or new preferred names were not collected. Overall, when asked if Pehchan had facilitated their access to these amenities, 4182 participants reported accessing social entitlement services with the support of the Pehchan programme by the end of July 2015.

### Self-efficacy and collective action

By the end of July 2015, a total of 2218 transgender-run community peer support groups were operational and the average time of participation by the end line survey was two years. In relation to self-efficacy, these data suggest that over half of these peer-support group members felt confident going to a health facility even if their identity was known, and over 63% felt confident getting tested for HIV at least once every six months at a government facility. Although this component was not collected in the baseline survey, results from the end line survey suggested high levels of confidence in collective action in cases of police arrest and intimate partner violence (Table 5).

**Table 5:** Self-efficacy and collective action of transgender respondents in the end line survey

Indicator	Proportion, %
Respondents who were willing to seek health services from a government clinic where health workers were aware of their transgender identity	58
Respondents who were confident that they would take an HIV test regularly (once in six months) at a government facility	63
Respondents who believed that “most or all” transgender people and hijra would work together to address a problem	74
Respondents who were confident that transgender participants or CBOs can speak collectively for the rights of transgender people	83
Respondents who were confident that transgender participants or CBOs can work together or keep each other from harm	79
Respondents who participated in a public event in the last six months where they could be identified as transgender	58
Respondents who were helped by other transgender participants or CBO when they were last arrested	61
Respondents who were helped by transgender peers or CBO when their partner became violent	46.4

CBO, community-based organization.

## Discussion

Our data demonstrate that the community-based and client-centric approaches of the Pehchan programme significantly increased access to a range of tailored health, HIV, psychological, social and legal services for transgender populations. By focusing on sexual minorities, the Pehchan programme could more effectively address the individual-level stigmas and interpersonal-level stigmas associated with the transgender population. While a corpus of evidence demonstrating that collectivization, empowerment and community mobilization are effective in increasing access to services for marginalized sexual minorities [34,36,42,43], studies among transgender populations are limited. This study makes a significant contribution to the literature in this regard. The key strength of this study is the inclusion of both programmatic and large survey data from significantly diverse districts and states, all of which demonstrate the positive impact of the programme.

The Pehchan programme relied on community involvement for the programme design and implementation. By using community-led feedback mechanisms, Pehchan mitigated some of the common concerns regarding operationalization of programmes that are based on a premise of community ownership, mobilization and agency [44], in which community involvement tends to be tokenistic, or simply not focused enough on specific minority groups to have an impact [34]. The Pehchan programme is unique in incorporating true community involvement at programme inception, design, implementation, administration and change over time.

While more detailed and longitudinal data related to the extent to which agency was increased would be useful, our survey respondents reported high levels of confidence individually and collectively in seeking services and acting collectively in defence of their rights. Evidence from other settings suggests that collective agency is associated with better access to STI services in MSM and transgender people in India [35] and increased condom use in sex workers in India [45]. Creation of peer support groups is an important avenue for enhancing collective efficacy and has been shown to increase positive behaviours in sex workers [46] and enhance the collective ability of people living with HIV to challenge and cope with stigma [47]. In conjunction with the limited previous studies, our data emphasize the importance of peer-support groups for transgender populations. Other scholars have also called for support groups for transgender populations [48], particularly given the synergistic impact that poor resilience, coping and lack of social support have on sexual risk among transgender individuals [10].

Experience from Pehchan suggested an evolution of the utility of collectivization approaches. Initially there was a high need for visibility, which was achieved through peer support groups and events such as “Hijra Habbas” (literally meaning “coming together of hijras,” with a festive connotation). These collectivization platforms later served as advocacy and convening platforms where the transgender people and hijras were able to secure an audience with and express their situations to important stakeholders, including the Minister of Social Justice and Empowerment, Secretary of Health, Commissioner of Human Rights, and representatives from law enforcement, NACO, UNAIDS, and other development partners.

Another important aspect of the Pehchan programme interventions is related to the differentiation of hijra from the transgender communities, both in terms of programmatic focus as well as socio-economic context. While these communities are often assumed to share a common social identity and community practices, the hierarchy within the hijra system works as a potential obstacle to the self-determination and autonomy of individuals within the system [49]. Thus, treating them as distinct communities in terms of identity and sexual risks was a central part of the programme.

At the structural level, our findings have important implications for informing how health systems can be strengthened to ensure access to a range of services specific to the transgender population needs, such as services related to sex reassignment and biological feminization, including hormone use and removal of the penis and/or testes. Given the convergence of multiple issues among transgender populations such as poor mental health, STIs, HIV, alcohol use and gender violence, appropriate health interventions should be a priority for health systems and services. Experience from our programme indicates that even simple operational procedures were a challenge to healthcare providers, who sometimes did not know to which ward they should admit or refer transgender females. Similarly, our findings suggest that social security and public entitlement programmes need to increasingly focus on ensuring equitable access to services by transgender populations.

While our results suggest that the intervention had positive impact, we note that, in keeping with minority stress theories, optimal impact of services for marginalized populations is often achieved when services are combined with policy interventions [50]. While inclusion of legal support mitigated structural factors of vulnerability of transgender clients, changing the legal and legislative framework to decriminalize sexual practices among transgender populations would contribute to reducing their exposure to violence and harassment.

In interpreting the findings, the limitations of our study should be noted. As indicated in some of the results, not all participants responded to all the questions. In addition, the generalizability of our findings to other settings might be limited due to differences in culture, epidemiology, religion and other social aspects. Nevertheless, the study provides useful information related to the provision of tailored, community-based services. Because the Pehchan programme was not providing all potential services, some of the data collected were about referral rather than actual utilization of services. Data related to collective efficacy were not collected in the baseline survey, which limits our ability to quantify the intervention. However, these findings form a starting point on which further research can be built. We acknowledge the lack of application of regression methods to limit confounding of observed effects. However, our analysis was primarily aimed at highlighting differences between baseline and end line samples, rather than identifying independent predictors of such changes. Finally, the baseline and end line samples did not constitute the exact same participants. This is a limitation of the extent to which individual-level impact can be claimed, as it introduces potential for confounding and artefactual impact resulting from sampling variation. However, our sampling from larger transgender communities served by the programme provides useful data related to the efficacy of the programme in those communities.

## Conclusions

The community-based, client-centric and gender-affirming approaches employed by the Pehchan programme significantly improved the demand for and access to tailored health, HIV, psychological, social, and legal services for transgender people. Community involvement and collectivization approaches strengthened self- and collective efficacy and identity of transgender communities and are greatly needed to address the unique needs of this marginalized group. Furthermore, structural changes in the legal and legislative framework to decriminalize sexual practices among transgender populations are a priority. The global 90-90-90 goals of controlling the HIV epidemic [25] cannot be achieved unless the HIV and health needs of transgender populations are addressed through an inclusive, rights-based approach.
